# Rapid evolution of ecological sexual dimorphism driven by resource competition

**DOI:** 10.1111/ele.14140

**Published:** 2022-11-10

**Authors:** Stephen P. De Lisle

**Affiliations:** ^1^ Evolutionary Ecology Unit, Department of Biology Lund University Lund Sweden

**Keywords:** *Drosophila melanogaster*, ecological character displacement, experimental evolution, nutritional geometry, resource competition, sex‐specific selection

## Abstract

Sex differences in ecologically important traits are common in animals and plants, and prompted Darwin to first propose an ecological cause of sexual dimorphism. Despite theoretical plausibility and Darwin's original notion, a role for ecological resource competition in the evolution of sexual dimorphism has never been directly demonstrated and remains controversial. I used experimental evolution in *Drosophila melanogaster* to test the hypothesis that resource competition can drive the evolution of sex differences in diet. Following just three generations of adaptation, offspring from flies evolved in low‐resource, high‐competition environments show elevated sexual dimorphism in diet preference compared to both the ancestor and populations evolved on high‐resource availability. This increased sexual dimorphism was the result of divergence in male sucrose intake and female yeast intake consistent with the differential nutritional requirements of the sexes. These results provide the first real‐time direct evidence for evolution of sexual dimorphism driven by resource competition.

## Graphical abstract

In an evolution experiment, I find that sexual dimorphism in diet evolves rapidly in response to resource competition, suggesting a role for ecology in driving the evolution of sexual dimorphism.

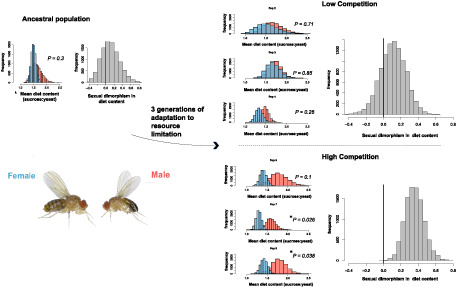

## PEER REVIEW

The peer review history for this article is available at https://publons.com/publon/10.1111/ele.14140.

## INTRODUCTION

Trait differences between the sexes, or sexual dimorphisms, are a striking source of diversity in sexually reproducing organisms (Andersson, 1994; Darwin, 1871; Fairbairn, 2013). Although these sexual dimorphisms often reflect traits related to mating, in many cases males and females have also diverged in traits related to ecological niche, such as body size, diet content, habitat use and feeding morphology (Hedrick & Temeles, 1989; Shine, 1989). This diversity of sexual dimorphism prompted Darwin (1871) to propose, in addition to sexual selection, the possibility of an ecological cause of sexual dimorphism (Hedrick & Temeles, 1989). In modern terms, this ‘ecological cause’ has been framed as a potential role for resource competition in driving ecological character displacement between the sexes (Slatkin, 1984). Mathematical models suggest character displacement between the sexes can readily evolve (Bolnick & Doebeli, 2003; Cooper et al., 2011; Li & Kokko, 2021; Slatkin, 1984), and a number of studies in birds (Temeles et al., 2000), salamanders (De Lisle & Rowe, 2015), lizards (Butler et al., 2007; Pincheira‐Donoso et al., 2018; Schoener, 1977) and other systems (De Lisle, 2019) provide evidence of an ecological cause of sexual dimorphism and suggest that resource competition may play a role. However, unlike other causes of the evolution of sexual dimorphism such as sexual selection, resource competition as an ecological cause of sexual dimorphism remains controversial and lacks unambiguous evidence (De Lisle, 2019), despite widespread effort (Arbuthnott et al., 2014; Fryxell et al., 2019; Giery & Layman, 2019; Gomez‐Llano et al., 2018; Perry et al., 2017; Svensson, 2019) to understand sex differences in an ecological context. We thus lack a direct demonstration that resource competition can drive evolution of ecological sexual dimorphism.

Laboratory experimental evolution provides a powerful approach for testing the hypothesis that ecological conditions may drive the evolution of sexual dimorphism. In *Drosophila* flies (Camus et al., 2017; Garlapow et al., 2015; Jensen et al., 2015; Reddiex et al., 2013) and other insects (Maklakov et al., 2008), male and female fitness are typically maximised under different ratios of protein and carbohydrate (Raubenheimer & Simpson, 1997), with female fecundity maximised under relatively high protein diets and male survival and mating success maximised under relatively high carbohydrate in no‐choice feeding experiments. However, in food choice experiments from multiple *Drosophila melanogaster* populations, standing variation in adult male and female diet preference remain largely overlapping, with both sexes remaining displaced from their fitness optima even when substantial sexual dimorphism exists (Camus et al., 2018; Reddiex et al., 2013), or in other cases, sexual dimorphism in diet remains insignificant (Jensen et al., 2015), or depends on ecological conditions (Davies et al., 2018; Lee & Kim, 2013). These past results imply that ecological conditions may determine the extent to which sexual dimorphism in diet preference evolves. Resource competition at the larval stage has been shown to be an important driver of adaptation to resource use in *D. melanogaster* (Bolnick, 2001). Thus, *D. melanogaster* makes an ideal model system to experimentally test the hypothesis that resource competition at the adult stage can drive character displacement between the sexes, with the a priori prediction that males and females will alternatively evolve elevated carbohydrate and protein source consumption, respectively, under elevated resource competition.

I evolved 6 replicate *D. melanogaster* populations under conditions of elevated adult resource competition to test the hypothesis that resource competition can drive the evolution of increased ecological sexual dimorphism. By housing flies in vials where larval food was covered with a thin layer of agar (readily penetrated by hatched larvae but not by adult flies; see Materials and Methods), I was able to manipulate adult liquid diet independently of larval diet using microcapillary tubes. This approach allowed me to assess the effects of competition at the adult stage, where sex differences are typically expressed, on the evolution of sexual dimorphism while holding larval ecological conditions constant.

## MATERIALS AND METHODS

### Fly rearing and experimental design

Experimental populations were derived from a LH_m_ stock population of *D. melanogaster*. This population is from a lab‐adapted population originally collected in California in 1991 from 400 females (Lund‐Hansen et al., 2020; Rice et al., 2005), and obtained by me from Jessica Abbott's lab group at Lund University, where their population has been maintained for over 500 generations. This population was maintained under conditions of 12:12 light, approximately 25 C, 60% humidity and fed standard fly medium consisting of cornmeal, yeast and molasses. As a standard component of the rearing protocol, yeast is typically added for consumption by newly eclosed adults to stimulate fecundity; thus, the population is adapted to high resource availability at the adult stage. One generation prior to the start of the experiment, diet preference was assayed by placing 50 males and 50 females in individual fly vials containing 5 ml of agar solution (to prevent desiccation) along with two 5 μl microcapillary tubes, one containing 90 g/L sucrose solution and the other containing 90 g/L yeast extract solution (ultrapure bacteriological grade, VWR J850). Flies were removed after 24 h and the length of fluid remaining in the microcapillary tubes was measured to the nearest hundredth of a millimetre with digital callipers.

Eight populations were established with seven 28‐mm‐wide fly vials each, with eight males and eight females per vial (16 flies total per vial), for a total of 112 flies per population (Table S1). Vials contained standard fly food (approx. 15 ml) to which the LH_m_ population is highly adapted, capped with a thin layer (approx. 3 ml) of agar solution to block access of adult flies to nutrition present in the larval food source. Larvae can readily penetrate this agar cap; a pilot experiment showed that the effects of the larval cap on vial reproductive output were minimal (mean eclosed from agar‐capped vials following oviposition by five females: 117, regular vials: 130, *F*_1,8_ = 1.66, *p* = 0.23). Microcapillary tubes were added to the vials for 48 h to provide a source of carbohydrate and protein for adult flies. After 48 h, adults and microcapillary tubes were removed from the vials and resulting offspring were allowed to develop. This process was then repeated in the next generation; at each generation, newly eclosed flies were removed from vials, pooled by population, counted, sexed and allocated randomly under light C0_2_ anaesthesia to new agar‐capped vials. Density was kept constant at 16 flies per vial, and population size was allowed to expand by expanding the number of vials in a population up to the founding size of seven vials. Four ‘high’ resource availability populations received 30 μl of sucrose solution and 30 μl of yeast solution (both 90 g/L) in a total of six 10μl microcapillary tubes. Four low resource availability populations received a single 5 μl microcapillary tube each of sucrose solution and yeast solution. These treatment levels were designed to achieve an environment characterised by high resource competition, and a comparator environment where resource competition was less severe *relative to the other treatment*; both environments were likely an increase in the extent of resource competition relative to the ancestral population where food was essentially available ad libitum. One population from each treatment group failed to produce enough flies in the first generation to yield a single vial at the standard density and so were treated as extinction events.

After three generations of adaptation to these environments, experimental flies were placed in regular food vials after removal from their agar‐capped experimental vials to produce offspring reared in a common environment for diet assay. These offspring (50 males and 50 females per population) were collected upon eclosion and placed individually in vials with 5 ml agar solution to prevent desiccation. Flies were held for 48 h after which surviving flies received two 5 μl microcapillary tubes, one containing 90 g/L sucrose solution and the other containing 90 g/L yeast extract solution. Twenty additional vials, lacking flies, were dispersed throughout the environmental chamber and received microcapillary tubes in order to control for effects of evaporative loss. Flies were removed after 24 h and the length of fluid remaining in the microcapillary tubes was measured to the nearest hundredth of a millimetre with digital callipers.

The stopping point of three generations for the diet and mortality assay was predetermined in advance; offspring counts after a fourth generation of treatment exposure were conducted to obtain recruitment data needed for a mean fitness measure for generation three, it was not possible to further assay diet due to logistical constraints.

### Controlling for evaporation

Evaporative loss was high in this experiment; past experiments using liquid media consumption from microcapillary tubes to measure diet in *D. melanogaster* have used very high relative humidity (>80%, [Reddiex et al., 2013; Camus et al., 2017]), which was not desirable in this design because (1) it would conflate the diet change of the experimental manipulation with a significant increase in relative humidity, since the ancestral population is adapted to 60% relative humidity, and (2) adaptation to desiccation risk in *D. melanogaster* entail changes in sex‐specific lipid and carbohydrate acquisition and storage and so diet itself is expected to be fundamentally tied to the humidity environment to which a population is adapted (Chippindale et al., 1998). I took two approaches to correct for evaporative loss. First, in the analysis of sexual dimorphism in the ratio of sucrose: yeast consumption, I corrected raw ratios by the ratio of evaporative loss from control vials, as (sucrose_raw_:yeast_raw_ – sucrose_evaporation_:yeast_evaporation_) + 1; equivalent conclusions were obtained using the raw values. For the analysis of bivariate sucrose and yeast consumption, in which negative values for some small number of individuals (arising due to measurement error) do not pose the conceptual or mathematical problem that they do for ratios, I subtracted sucrose_evaporation_ and yeast_evaporation_ from the respective raw values. Corrected values for all populations were significantly greater than zero (all *p* ≤ 0.00017), indicating that this measure of fluid loss is indeed capturing consumption of individual flies; equivalent general conclusions were obtained in the analysis of the raw length of fluid remaining. Moreover, the final corrected measures of 24 h food consumption in my experiment are consistent with those of previous studies of microcapillary feeding in *D. melanogaster* (Camus et al., 2017; Lee et al., 2008).

### Statistical analysis

**FIGURE 1:**
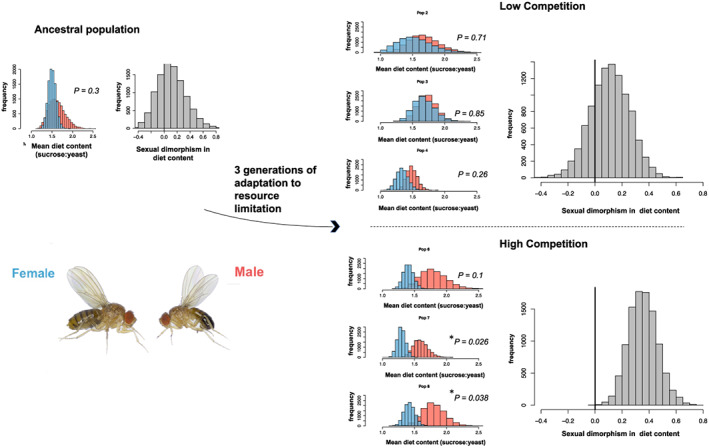
Sexual dimorphism in diet content evolved from a monomorphic ancestor in three generations. Sampling distributions of male (red) and female (blue) mean sucrose: yeast consumption ratios show large overlap between male and female diet in the ancestral population (left panel), and sexual dimorphism was not statistically significant (sampling distribution of the difference in male and female mean shown). After three generations of adaptation to high competition (low resource availability), two populations showed significant sexual dimorphism and the third replicate was trending towards significant dimorphism. On average, populations adapted to high competition showed significant sexual dimorphism, with males consuming higher sucrose: yeast ratios than females (right panel, bottom). Sexual dimorphism was not significantly different from zero in populations adapted to low competition, high resource availability (upper right). *p*‐values are from linear models with sex as a main effect and sucrose: yeast ratio as the response, fit separately for each population. Sampling distributions were calculated by bootstrapping, stratified by population for pooled sampling distributions of the difference.

I used separate linear models for each population to assess the effect of sex on the ratio of sucrose: yeast consumption, to infer the significance of sexual dimorphism in diet content on a population by population level. To assess treatment effects on diet content, I used a mixed model with mean sucrose: yeast ratio for each population*sex combination as the response, and sex, treatment and their interaction as fixed effects. This model included population as a random effect to account for nonindependence between male and female means from the same population, and also accommodated uncertainty in mean sucrose: yeast ratios by modelling the variation in each mean (obtained from a bootstrap of 10,000×) as a fixed error variance of each observation. I analysed population mean sucrose: yeast because population is the unit of replication and because taking means of the ratios avoids challenges associated with non‐normality of raw individual feeding ratios (due to the central limit theorem). Similar qualitative conclusions were obtained in analyses of individual‐level data. To illustrate population‐level sexual dimorphism and treatment‐wide effects shown in Figure 1, I obtained bootstrapped (10,000×) sampling distributions for sexual dimorphism in sucrose: yeast for each population, as well as overall sexual dimorphism in each treatment group (for later by bootstrap via stratified, by population, random sampling with replacement, 10,000X; two‐tailed p‐values were obtained directly from these sampling distributions). To assess mortality effects, I fit a binomial glm with each populations' survival and mortality as the response. To analyse bivariate response in sucrose and yeast consumption, I fit a series of Bayesian multiresponse mixed effects models with varying degrees of complexity; a model with just trait‐specific intercepts, a model with treatment effect, sex effect, treatment*trait+sex and sex*trait + treatment. All models contained a random trait‐specific intercept among populations and an unstructured residual covariance matrix; default priors were used. Of these models, the model with sex*trait interaction and main effect of treatment fit best (ΔDIC = 2.7). All statistical analyses were performed in R; linear models were fit using lm(), mixed effects models using lme() and multiresponse mixed effects models were fit using MCMCglmm (Hadfield, 2010). Complete R script is provided as supporting material.

## RESULTS

Sexual dimorphism in diet preference in the ancestral population was weak and not statistically significant (male sucrose: yeast—female sucrose: yeast = 0.113, *SE* = 0.22, bootstrapped *p* = 0.6, linear model *p* = 0.3). At the conclusion of the survival assay following three generations of adaptation, diet preference data were obtained from 439 flies (240 low food availability, high competition; 199 high food availability, low competition). After just three generations, offspring from all populations evolving under high competition (low resource availability) showed elevated sexual dimorphism in sucrose: yeast consumption, with males showing increased sucrose: yeast ratios compared to females (Figure 1; male sucrose: yeast—female sucrose: yeast = 0.356, *SE* = 0.108, *p* = 2 × 10^−4^) consistent with predictions. This sexual dimorphism was statistically significant or trending towards significance in all three replicate populations (Figure 1). Sexual dimorphism was weak, similar to the ancestral state and not statistically significant in populations evolving under low competition (male sucrose: yeast – female sucrose: yeast = 0.109, *SE* = 0.143, *p* = 0.437); this treatment effect on sexual dimorphism was statistically significant (linear mixed model, *F*_1,4_ = 25.3, *p* = 0.0073). Observed sucrose: yeast ratios were consistent with past studies of *D. melanogaster* and indicated consumption of sucrose: yeast in an approximate 1.5:1 ratio (Ancestor sucrose: yeast, M = 1.62, F = 1.50; High competition sucrose: yeast M = 1.73, F = 1.37; Low competition sucrose: yeast M = 1.63, F = 1.52).

**FIGURE 2:**
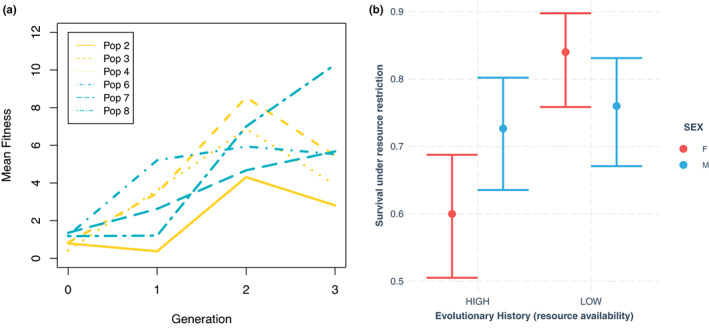
Populations adapted to environmental conditions over the course of the experiment. Panel (a) shows mean fitness (calculated from recruitment per female to the next generation) of each population from the onset of the experiment; mean fitness increased for all six populations over the course of the experiment. Panel (b) shows survival (estimates and 95% CIs from a binomial glm) during 48 h of resource restriction in offspring from generation 3 flies. Populations adapted to low resource availability (high competition) showed elevated survival under resource restriction compared to flies evolved under high resource availability.

**FIGURE 3:**
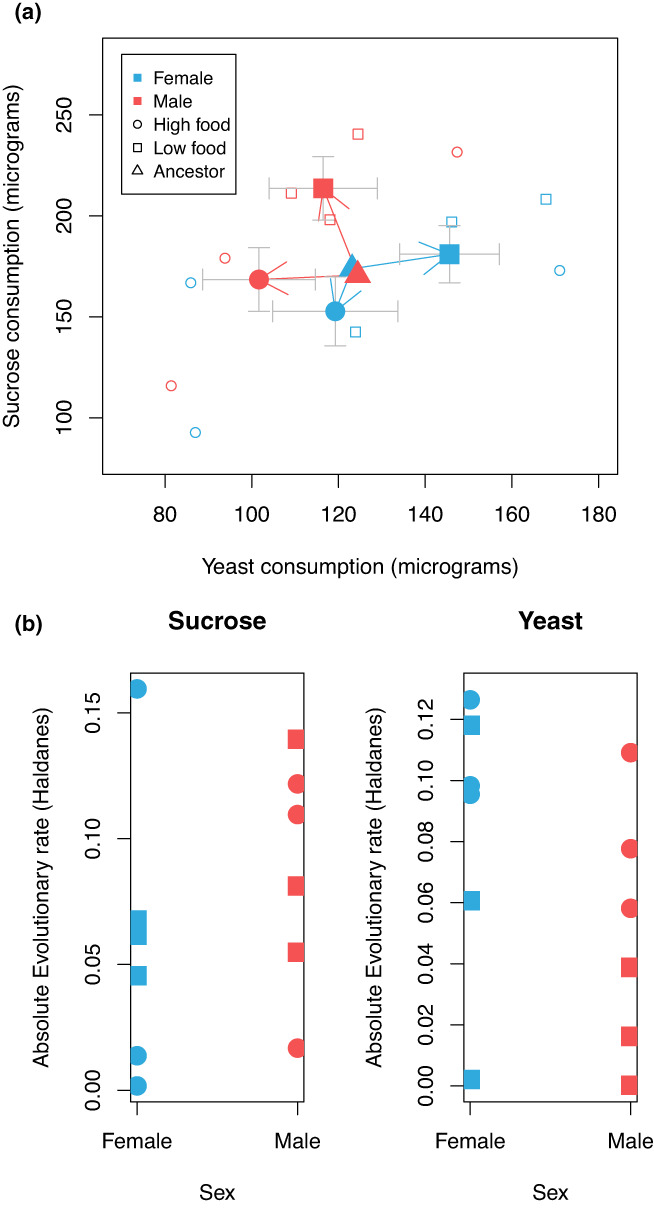
Evolution of sexual dimorphism occurred via divergence in both male and female diet. Panel (a) shows mean sucrose and yeast consumption for offspring from males and females from each population (small open markers) after three generations of adaptation, as well as the treatment means (large filled markers; error bars show bootstrapped SEs). Arrows connect male and female ancestral means to the corresponding means for populations adapted to high and low resource availability. Panel (b) shows the absolute value of the evolutionary rate in Haldanes for each sex for each population, measured as 1/3 of the difference between generation 3 mean diet content and the ancestral mean over the ancestral standard deviation; squares are high competition, low food, circles are low competition, high food.

Mean absolute fitness, measured as recruitment per female, of all six populations increased an order of magnitude during the course of the experiment, indicating evolution was adaptive (Figure 2a). Observed fecundity values observed from 2‐day windows of oviposition in this experiment were consistent with daily oviposition rates observed in other studies of *D. melanogaster* reared on an adult liquid diet of similar caloric content (Lee et al., 2008). Survival during 48 h of food deprivation was higher for offspring from high competition, low food environments than for flies evolving in high food conditions (generalised linear model *Z* = 4.5, *p* < 0.0001, Figure 2b), indicating that high competition populations evolved adaptation to resource restriction. An analysis of bivariate diet content (Raubenheimer & Simpson, 1999) revealed that populations evolved under resource restriction evolved elevated levels of resource consumption compared to high resource populations (bivariate mixed effects model, posterior mean treatment effect = 25.9 [3.7–47 95% CI] micrograms, *pMCMC* = 0.034; Figure 3a), indicating adaptation to elevated competition. Males evolved elevated sucrose intake while females evolved elevated yeast intake (bivariate mixed effects model, posterior mean trait*sex = −48 [−84 to −13 95% CI] micrograms, *pMCMC* = 0.014; Figure 3a), and evolutionary rates were not significantly different between the sexes (sucrose: linear model *F*_1,10_ = 0.96, *p* = 0.34, yeast: *F*_1,10_ = 1.8, *p* = 0.21, Figure 3b), indicating that evolution of sexual dimorphism in diet was not driven by divergence in one sex, but rather by divergence in both male and female diet.

## DISCUSSION

Sexual dimorphisms in ecological niche and related traits, although less striking than sexual dimorphisms in mating and display traits (Li & Kokko, 2021), are nonetheless apparent in many animals (Shine, 1989). These ecological sexual dimorphisms have led to the hypothesis that resource competition can, on its own (Rand, 1952; Slatkin, 1984) or in concert with sexual selection (De Lisle, 2019), contribute to generating sex‐specific selection and thus drive the evolution of sexual dimorphism. However, unequivocal evidence for resource competition as a driver of sexual dimorphism is scant, primarily due to lack of experimental empirical tests (De Lisle, 2019). Here, I used experimental evolution in *Drosophila melanogaster*, and found that increased sexual dimorphism in diet evolved rapidly in response to elevated adult resource competition, while male and female diet content remained similar to ancestral composition in a low competition treatment. These results provide direct evidence that resource competition contributes to the evolution of sexual dimorphism.

Theory suggests that competition can drive sexual dimorphism in resource use most readily when the sexes interact in small demes with reliably distributed resources (Li & Kokko, 2021). The experiment reported here is consistent with this expectation in that the design entailed interactions occurring in demes (vials) of eight males and eight females, with consistent spatial distribution of resources across vials and generations within a population. Although male‐specific displacement is expected under conditions of monogamy (Li & Kokko, 2021), *D. melanogaster* are not monogamous (Markow, 1996), and the finding of displacement in both sexes is thus consistent with my experimental design and predictions of character displacement theory.

The predictable direction of the evolution sexual dimorphism under high competition (elevated male sucrose intake, elevated female yeast intake) in this experiment indicates that resource competition acts along with nutritional requirements related to sex‐specific reproductive strategies to influence male and female diet evolution (De Lisle, 2019). In flies and other insects, females face a physiological requirement for protein in order to reproduce, while male reproductive success depends less‐fundamentally on access to protein (Camus et al., 2017; Lee et al., 2008; Maklakov et al., 2008). The evolution of elevated sexual dimorphism in response to resource competition observed in the present study indicates that resource competition can contribute to, or enhance, sex‐specific diet evolution. However, it is important to consider that, as noted above, fly diet is fundamentally tied to male and female reproductive requirements; the data presented here cannot indicate competition as a sole cause of the evolution of sexual dimorphism in diet. Alternative experimental approaches would be required to test for a role of resource competition in driving sexual dimorphism independently of reproductive roles, although such designs are not straightforward (also see below). In any case, resource competition seems most likely to play a role in furthering the evolution sexual dimorphism initiated due to divergent reproductive strategies (De Lisle, 2019), as the results of this experiment indicate, as opposed independently driving evolution of de novo sexual dimorphism.

Parameter estimates for male and female resource acquisition in all replicate populations were generally consistent with past studies of *D. melanogaster*. Mean sucrose: yeast consumption ranged from 1.37 to 1.73, corresponding to carbohydrate: protein ratios (Lee et al., 2008) of approximately 3:1–4:1, with consistently elevated sucrose: yeast for males relative to females in a population. These values are generally consistent with past work showing carbohydrate: protein ratios ranging between 2:1 and 4:1 in choice experiments in *D. melanogaster* (Camus et al., 2018; Jensen et al., 2015; Lee et al., 2008; Reddiex et al., 2013). Past studies of male and female fitness under no‐choice food manipulations (Camus et al., 2017; Reddiex et al., 2013) indicate that sex‐specific fitness is maximised under more extreme carbohydrate: protein ratios than observed in the present experiment or in other populations, suggesting that while competition can contribute to the exacerbation of sexual dimorphism, it did not drive the sexes to their hypothesised optima in just three generations.

A growing number of studies have employed experimental evolution to understand the effects of various forms of competition on evolution in real time (reviewed in Rodrigues et al., 2016), yet relatively few studies have used this approach to explore the link between intraspecific resource competition and niche evolution (Bolnick, 2001; Bono et al., 2012). To my knowledge, no prior studies have examined the potential role of ecological sexual dimorphism as an evolutionary response to resource competition in an experimental context. Yet, in sexually reproducing taxa, sex‐specific responses to competition may play a major role in any observed competition‐driven niche expansion. For example, character displacement theory suggests that the time lag expected to observe an evolutionary response to competition can be substantial (Doebeli, 1996; Schluter, 2000), yet this lag may not be expected when other forms of selection are acting in concert with competition, as is expected in the cases of the sexes (De Lisle, 2019).

A major caveat in this study is that I cannot exclude the possibility that variation in the strength of sexual selection or fecundity selection has contributed to the observed evolutionary response. Although density and sex ratio were constant across treatments, it is possible, for example, that differences in mean condition between low and high resources availability environments could contribute to generating differences in the strength of condition‐dependent sexual selection (De Nardo et al., 2021; Delcourt & Rundle, 2011). Future studies that add in factorial complexity would be informative, although alternative designs where mating system is manipulated independently (Rundle et al., 2006) of resource availability carry their own interpretive challenges; in particular the difficulty of manipulating mating system without also manipulating total density (and thus the strength of competition). A second caveat is that although adult density was constant across treatment and time in this experiment, larval density was likely not. However, given that adult recruitment was very similar between low‐ and high‐competition populations (Figure 2a), it is unlikely that differences in larval density can explain differential responses observed across the treatments.

This experiment demonstrates that an environment of elevated resource competition can drive the rapid evolution of increased ecological sexual dimorphism from a less‐dimorphic ancestor, as one component of adaptation to elevated adult resource competition. Although these results are under laboratory conditions in a model organism, evolutionary rates of sucrose (0.072 Haldanes, average) and yeast (0.066 Haldanes, average) consumption in this experiment (Figure 3b) are well within the range observed in contemporary studies of microevolution in the wild (Hendry & Kinnison, 1999). Combined with the prevalence of ecologically important sex differences from a diverse range of taxa (De Lisle, 2019; Shine, 1989; Temeles et al., 2000), evidence from natural systems such as competition‐driven divergent selection (De Lisle & Rowe, 2015) and geographic patterns consistent with character displacement between the sexes (Butler et al., 2007; Pincheira‐Donoso et al., 2018), and functional morphology studies (Temeles et al., 2000), the experiment presented here provides a proof‐of‐concept that demonstrates that Darwin's idea of an ecological cause of sexual dimorphism may be an important and general contributing cause to the diversity of sexual dimorphism observed in nature.

## AUTHOR CONTRIBUTION

S.P.D. was the sole contributor to all aspects of this manuscript.

## Supporting information


Table S1
Table S2

## Data Availability

All data are archived on Dryad doi: 10.5061/dryad.j3tx95xjh.
