# Supplementary material for: Rapid evolution of ecological sexual dimorphism driven by resource competition
Source: Ecol Lett. 2022 Nov 10;26(1):124–31. doi: 10.1111/ele.14140 (PMC10099664; doi:10.1111/ele.14140)
Supplement: Supplementary file 1 — Table S1 Table S2 [file ELE-26-124-s001.pdf]

Supplementary Material

Table S1. Summary of Population Sizes at each Generation

Generation 0

population	N	vials
1	112	7
2	112	7
3	112	7
4	112	7
5	112	7
6	112	7
7	112	7
8	112	7

Generation 1

population	N eclosed	vials for next generation
1	6	0
2	89	5
3	47	3
4	23	1
5	0	0
6	68	3
7	76	4
8	66	3

Generation 2

population	N eclosed	vials for next generation
2	30	1
3	83	5
4	29	1
6	125	6
7	84	5
8	29	1

Generation 3

population	N eclosed	vials for next generation
2	69	4
3	342	7
4	55	3
6	285	7

7	187	7
8	56	3

Generation 4

population	N eclosed	vials for next generation
2	180	NA
3	305	NA
4	94	NA
6	310	NA
7	318	NA
8	247	NA

Table S2. Summary of diet content by sex and population

Sex	Population	Treatment (Food amount)	Mean Sucrose (mg)	SE	Mean yeast (mg)	SE	mean sucrose:yeast	SE	N
F	2	HIGH	92.69	37.0	86.99	37.6	1.54	0.263	20
M	2	HIGH	115.83	26.0	81.42	25.1	1.68	0.230	39
F	3	HIGH	166.85	27.0	85.93	24.0	1.68	0.165	35
M	3	HIGH	179.04	23.7	93.82	21.0	1.72	0.149	45
F	4	HIGH	172.95	27.9	171.02	17.4	1.34	0.090	35
M	4	HIGH	231.59	32.5	147.36	22.6	1.49	0.085	25
F	6	LOW	196.97	28.4	146.11	21.4	1.40	0.079	36
M	6	LOW	211.27	25.4	109.14	21.5	1.79	0.204	41
F	7	LOW	142.50	24.9	123.94	19.0	1.30	0.065	46
M	7	LOW	198.10	23.6	118.05	19.2	1.59	0.115	44
F	8	LOW	208.25	27.0	167.86	16.5	1.42	0.083	44
M	8	LOW	240.48	32.4	124.52	24.0	1.79	0.177	29

36
